# Learning the Capacity to Feel Better Through Experiment in a Box (XB): Qualitative Interpretive Description and Prototheory Development of Tuning

**DOI:** 10.2196/85164

**Published:** 2026-09-21

**Authors:** Steven A De La Torre, Richard Gomer, Eric Hekler, m c schraefel

**Affiliations:** 1 Herbert Wertheim School of Public Health and Human Longevity Science, University of California, San Diego, San Diego, CA, United States; 2 School of Information, University of Michigan, 2200 Hayward Street, Ann Arbor, MI, 48109, United States, 1 734 763 2285; 3 School of Electronics and Computer Science, University of Southampton, Southampton, United Kingdom; 4 Design Lab, University of California, San Diego, CA, United States; 5 WellthLab, Electronics and Computer Science, University of Southampton, Southampton, United Kingdom

**Keywords:** digital health, qualitative research, mHealth, behavioral change, dietary habits, movement, exercise

## Abstract

**Background:**

Experiment in a Box (XB) is a guided, time-limited framework designed to help individuals test and adapt simple eating and movement heuristics within the context of everyday life. Rather than emphasizing external performance metrics alone, XB invites participants to evaluate practices through felt experience, including whether a practice helps them feel better, worse, or the same. Although prior XB studies have shown promise for supporting health behavior change, less is known about the experiential and interpretive processes through which participants use XB to connect felt experience with action, adaptation, and continued practice.

**Objective:**

This study aimed to develop a preliminary prototheory of tuning to explain how participants used felt experience to guide eating and movement experimentation within XB. We define tuning as a dynamic, person-centered process through which individuals learn to sense, interpret, and act on bodily and contextual feedback during guided experimentation.

**Methods:**

We conducted a secondary, post hoc qualitative inquiry using interpretive description, reflexive thematic analysis, and abductive analysis. The qualitative corpus consisted of responses to 39 open-text postintervention survey questions completed across 3 survey sections. Descriptive implementation information was used to characterize participant flow and survey completion. A total of 44 participants enrolled and accessed the XB app. Of these, 12 completed the general postintervention survey, 11 completed the MOVE survey section, and 8 completed the XB app and implementation section. Self-determination theory and activity theory were used as sensitizing frameworks rather than fixed coding templates. Analysis involved iterative coding, analytic memoing, conceptual mapping, and team-based review to develop a preliminary prototheory of tuning.

**Results:**

The analysis resulted in a prototheory of tuning organized around recursive loops linking action, felt experience, interpretation, adaptation, and future action. Participants described XB as creating a low-pressure structure for trying practices, noticing bodily and experiential signals, interpreting those signals through practical health heuristics, and adapting practices to their bodies, routines, social relationships, and environments. Over time, repeated experimentation appeared to support the development of portable knowledge, skills, and practices, including movement snacks, eating heuristics, and an ongoing experimental orientation toward health behavior. Social, material, and environmental conditions shaped whether and how participants could engage in tuning, particularly across home, work, and family contexts.

**Conclusions:**

Tuning offers a preliminary conceptual lens for understanding how guided experimentation may support health behavior change by helping participants connect action, felt experience, interpretation, and adaptation. Rather than positioning behavior change as compliance with external prescriptions or metrics, the tuning prototheory foregrounds how individuals may cultivate the capacity to notice, interpret, and act on felt experience in context. Future prospective qualitative and mixed methods studies should test, refine, and potentially challenge this prototheory across more diverse populations and implementation settings.

## Introduction

### Background

Digital health tools often support health behavior change through external prompts, goals, reminders, self-monitoring, and performance metrics. These approaches can be useful, but they may also position health behavior change as a process of responding to external feedback rather than learning from one’s own bodily and contextual experience. This distinction is important because interoception, or the capacity to sense and interpret internal bodily signals, is a key process individuals use to understand states such as fatigue, hunger, fullness, energy, discomfort, and recovery [1-3]. When individuals have difficulty sensing or interpreting these signals, they may have fewer experiential resources for determining whether a given eating or movement practice is useful in their own lives [4-8].

Embodied and inbodied interaction approaches offer an alternative orientation for health technology design by treating bodily experience, context, and action as central to how people engage with health practices [9,10]. From this perspective, digital tools should not only prescribe or track behavior; they should help people notice, interpret, and adapt practices in ways that align with their bodies and everyday contexts. This orientation is especially relevant for interventions that aim to support sustainable eating and movement practices, where the effectiveness of a behavior may depend on how it is experienced and adapted in daily life.

### Experiment in a Box and Tuning

Experiment in a Box (XB) was developed as a guided, time-limited framework for helping individuals test and adapt health heuristics in everyday life [11]. In XB, participants are invited to try short eating and movement experiments, reflect on how those practices feel, and adapt them based on their own experience. Rather than treating external metrics as the only valid source of feedback, XB foregrounds participants’ felt experience, including whether a practice helps them feel better, worse, or the same.

Prior XB work has described this process as tuning: a process through which individuals use felt experience to adapt health practices in ways that better fit their bodies, goals, and contexts [12]. However, tuning has not yet been articulated as a preliminary theory that explains how guided experimentation may help individuals move from trying a practice to sensing its effects, interpreting those effects, adapting future action, and developing portable knowledge, skills, and practices. Without such a theory, it remains difficult to explain how XB may work, what mechanisms future studies should test, and how digital health tools might be designed to support this process.

### Objective

The purpose of this paper is to develop a preliminary prototheory of tuning using secondary qualitative analysis of participant accounts from the Build Burn Better (BBB) 2023 implementation of XB. Specifically, we aimed to explain how participants described using felt experience to guide eating and movement experimentation, and how these experiences informed a recursive model linking action, felt experience, interpretation, adaptation, and future practice.

## Methods

### Study Design and Methodological Orientation

This study was conducted as a secondary, post hoc qualitative inquiry of participant accounts generated during the BBB 2023 implementation of XB. Because the aim was to develop a preliminary, practice-oriented account of how participants made sense of guided experimentation, we drew on interpretive description [13,14], a qualitative methodology developed for applied health disciplines to generate practically useful conceptual understanding. We combined this orientation with reflexive thematic analysis [15,16] and abductive analysis [17]. Reflexive thematic analysis was used to identify patterns of meaning across participant accounts, while abductive analysis supported iterative movement between the data, sensitizing theoretical constructs from self-determination theory (SDT) [18-21] and activity theory (AT) [22,23], and emerging conceptual insights. The analysis was not designed to produce a definitive grounded theory, estimate theme prevalence, or establish saturation. Rather, it aimed to develop a transparent and theoretically informed prototheory of tuning that can guide future empirical testing and intervention design.

We used the theory construction methodology (TCM) [24] as a broad guide for early-stage theory development. TCM describes theory development as a staged process that begins with identifying the real-world phenomenon to be explained and constructing an initial conceptual framework or prototheory, followed by later formalization, testing, and refinement. The present study focused on the first two stages: (1) identifying experiential processes reflected in participant accounts of XB and (2) constructing an initial prototheory of tuning. Later stages of theory formalization and empirical evaluation are beyond the scope of this paper.

In keeping with this approach, SDT and AT were used as sensitizing frameworks rather than fixed coding templates. These theories helped orient attention to motivation, autonomy, competence, relatedness, tools, actions, and contexts, while leaving space for concepts that did not fit neatly within each framework. Abductive analysis was used to move iteratively between participant accounts, existing theory, and emerging interpretations, with the goal of developing a plausible conceptual account of how guided experimentation may support tuning [25]. Unlike grounded theory approaches that typically avoid beginning with a predetermined interpretive framework [17], our abductive approach explicitly treated SDT, AT, and prior XB concepts as provisional thinking tools. This allowed us to bridge participants’ reported experiences with theoretical insights while iteratively developing and refining a prototheory of tuning.

### The XB Approach

XB [11] is a guided experimentation framework designed to help individuals test and adapt health heuristics in everyday life. In the BBB 2023 implementation, participants were invited to complete a 5-week series focused on eating and movement practices (Table 1). Each week introduced EAT and/or MOVE experiments that participants could try in their own contexts. The experiments were intentionally time-limited and framed as opportunities to learn from experience rather than as permanent prescriptions for behavior change.

Participants used the XB app to view weekly experiment guidance, complete daily task lists, and reflect on their experiences (Multimedia Appendix 1). Rather than emphasizing external performance metrics, XB invited participants to notice how practices felt and to report whether they felt better, worse, or the same. Weekly Microsoft Teams meetings and discussion spaces provided additional support for preparation, reflection, questions, and peer learning. Participants were not instructed to spend a predetermined minimum number of hours using XB. Instead, engagement was structured around trying the weekly EAT and MOVE experiments in daily life, completing brief app-based task check-ins and optional reflections, and participating in weekly group support when available. The present manuscript provides only the study details necessary to understand the qualitative analysis and resulting prototheory. Further details on the XB framework and its design rationale have been reported previously [11].

**Table 1.** Components of the Build Burn Better 2023 XB^a^ implementation*.*

XB component	Participant activity	Support provided
Weekly experiment guidance	Review EAT and MOVE experiment instructions	XB app and weekly Teams meeting
Daily task list	Try experiment-related practices and indicate completion	XB app task prompts
Daily/weekly reflection	Reflect on felt experience, including whether practices felt better, worse, or the same	XB app reflection prompts
Microsoft Teams discussion	Ask questions, share experiences, and review resources	Investigator and peer support
Weekly group session	Prepare for upcoming experiments and discuss prior experiences	Investigator-facilitated Teams session

a XB: Experiment in a Box.

### Setting and Implementation Context

The BBB Summer Series 2023 was a 5-week implementation of XB offered to University of Southampton staff interested in exploring eating and movement practices to support feeling better. The series was deliberately framed around feeling better rather than weight loss or body composition. Recruitment materials were distributed through participating group managers, the Living Lab website, Teams channels, digital newsletters, and Staff Wellbeing Champion communications. BBB participants used the XB app and optional Teams-based support to engage with weekly EAT and MOVE experiments.

### Participants, Recruitment, and Sampling

Participants were recruited from University of Southampton staff during the BBB 2023 summer series with the final sign-up date of June 28, 2023. The series began on July 6, 2023, and ran for 5 weeks. Recruitment occurred through channels including email, the Living Lab website, and digital newsletters. The target population was university staff interested in exploring eating and movement practices to support feeling better. Participation was voluntary. A total of 44 participants enrolled and accessed the XB app. Of these, 12 completed the general postintervention survey, 11 completed the MOVE survey section, and 8 completed the XB app and implementation section. These postintervention surveys provided the primary qualitative experience data analyzed in this manuscript.

Because the present analysis was conducted as a secondary, post hoc qualitative inquiry using data generated during a real-world implementation, detailed information about study or recruitment nonresponders and reasons for refusal was not systematically collected. We therefore report available participation and engagement data and treat nonresponse as an important limitation for interpreting transferability. No systematic feedback was collected from participants who did not complete the exit survey, and reasons for exit survey noncompletion were also not available.

### Data Collection

In the week following completion of the BBB series, participants were invited to complete a postintervention survey through the XB app. The survey included open-text questions about participants’ experiences with XB, the guided experimentation process, and the EAT and MOVE experiments. These open-text responses served as the primary qualitative corpus for this analysis. The survey contained 39 open-text questions. The questions were presented in 3 sections: MOVE experiment experiences, general study experiences, and XB app and implementation experiences. The questions were optional, and no minimum or maximum response length was imposed. These were completed without interviewers present, so no additional probing, requests for elaboration, or follow-up questions were asked. Responses ranged from brief evaluative statements to more detailed accounts of participants’ experiences, interpretations, adaptations, and intentions. Due to the optional nature of the surveys, not every participant provided answers to all questions. The exact wording of the open-text questions and number of responses captured is provided in Table 2.

As discussed, participants’ exit-survey responses about the XB app, Teams, and other implementation supports were included in the qualitative corpus because they captured participants’ perspectives on how intervention tools and contextual supports facilitated or constrained guided experimentation. However, the underlying XB app interaction logs, Teams message-board content, and recordings of weekly Teams sessions were not coded or analyzed as qualitative data and were not used as evidence for the reported themes or prototheory. Descriptive enrollment and app-access information was used only to characterize participation in the broader BBB implementation and was reported in the Participants, Recruitment, and Sampling section.

**Table 2.** Open-ended postintervention survey questions included in the qualitative analysis.

Open-ended survey question	Responses captured, n/N
MOVE experiment questions	
1. What are one or two things you learned from the Movement Experiments overall? You could write about liking a movement, something you learned about yourself, how it met any expectations—anything…	11/11
2. One or two thoughts about the gravity connection driving why we need to work on strength, mobility, balance, and coordination to move? If it connected with you at all, what does it help you think about?	10/11
3. Anything you would like to share about the concept of “finding fatigue” and how you used it during, or after, your movement practices?	9/11
4. We looked at BALANCE each week as an assessment. What are one or two things you learned from these assessments about how you feel on any given day, or about your movement practice?	10/11
5. What are one or two takeaways, insights, or thoughts from your experience trying the Animal Moves and sit-stand?	11/11
6. If you have not said so already, which, if any, of the animal moves, sit-stands, or pulls/hangs do you think you will keep in your future?	10/11
7. What are some of your insights or thoughts about movement snacks and how they contributed to your movement practice?	10/11
8. Any other thoughts you would like to share about the MOVEMENT experiments in this study?	7/11
General study-experience questions	
9. Based on your rating above, what are one or two things that you found helpful—or less helpful—about this experiment approach?	12/12
10. Overall, what are one or two things—anything at all—that, from your perspective, went well during the study?	12/12
11. What are some things that, from your perspective, did not go well during the study?	12/12
12. What are some things, if any, that you hoped to get from participating in the study?	12/12
13. And what are some things, if any, that you think you did get from participating?	12/12
14. What are some ways, if any, that participation in the study has had an impact, positive or negative, on things that are important to you?	11/12
15. We offered some heuristics about eating during the study. Which ones do you think you may keep using?	12/12
16. What are some reasons for you continuing, or not continuing, to use those EAT heuristics?	12/12
17. Do you have any particular MOVE or EAT aspirations or related goals that you would like to achieve over the next 12 months?	12/12
18. What are any other concepts that have stuck with you that you learned or discovered during the study—whether MOVE, EAT, or both—that you might continue to use in your life?	11/12
19. For whatever your aspirations or goals involving MOVE or EAT, how has your experience here helped develop a better foundation for that aspiration?	8/12
20. If you have not shared this already, and if you have any goals or wishes, what are some reasons that those things are important to you?	9/12
21. And, if you have any goals or wishes, do you think that continuing with anything that you learned or practiced during the study will help you to achieve those things?	8/12
22. From what you have said about the things that are important to you, what do you think are some ways that continuing with any of the practices you learned during the study may have an impact on those things?	7/12
23. Finally, is there anything else that you would like to tell us about your experience of participating in the study?	7/12
XB^a^ app and implementation-experience questions	
24. Did you encounter any challenges while navigating through different features? If so, please describe them.	8/8
25. Were there any specific aspects of XB that you found particularly user-friendly or cumbersome?	7/8
26. Did XB’s features adapt well to different environments for the experiments—MOVE or EAT ones—or were there any limitations?	7/8
27. What specific changes or enhancements would you suggest to improve XB’s functionality and overall user experience?	5/8
28. Were there any missing features or functionalities that you expected to find in XB?	5/8
29. Were there any aspects of XB or features that did not feel relevant to your goals, or to the things that you were being asked to explore?	5/8
30. Did you develop a strong connection or attachment to any specific features or elements of XB? If so, which ones, and for what particular reasons?	5/8
31. Were there any design elements that resonated with you emotionally?	6/8
32. Did XB effectively motivate and encourage you to maintain engagement with the study? What are one or two examples of that?	6/8
33. Were there any aspects of XB that you found particularly engaging or motivating?	5/8
34. Were there any areas where you felt XB could have provided more assistance or feedback?	4/8
35. Did you integrate XB with other fitness tools or devices? If so, how well did they work together?	5/8
36. Were there any compatibility issues you encountered when using XB with other tools?	5/8
37. Engagement with Teams: Did you find the material on Teams helpful to your engagement in the study?	8/8
38. What are your thoughts about why you might have stayed engaged with the study or why you were less engaged?	7/8
39. What are one or two things we might do next time with the presentation to make it better for you in terms of experience? Others will likely share your experience, so we really appreciate your ideas.	7/8

a XB: Experiment in a Box.

### Data Analysis

The qualitative corpus comprised all nonblank responses to the 39 open-text postintervention questions provided by up to 12 participants across the 3 survey sections. These responses were uploaded to ATLAS.ti (Mac, version 25.0.1; ATLAS.ti Scientific Software Development GmbH) for analysis. Descriptive counts were used to characterize enrollment, app access, and completion of the 3 exit-survey sections. These data were not integrated with the qualitative coding or used to evaluate intervention effectiveness. Responses to the XB app and implementation questions were analyzed as part of the same qualitative corpus rather than as a separate usability analysis. During coding, these responses were examined for how digital tools, relational supports, and implementation conditions facilitated or constrained participants’ experimentation, interpretation, and adaptation. This was consistent with the use of AT as a sensitizing framework for understanding tools, mediated action, and social and material conditions, and with the use of SDT to orient attention to competence, autonomy, and relatedness. Our analytic approach for the exit survey open-text experience data was rooted in concepts related to the TCM approach described above [24].

The primary qualitative analysis proceeded in 4 stages. First, the first author reviewed the open-text survey responses for familiarization and wrote analytic memos focused on participants’ descriptions of guided experimentation, felt experience, adaptation, and continued use of XB-related practices. Second, initial codes were developed using an abductive approach. SDT, AT, and prior XB/tuning concepts were used as sensitizing frameworks, while codes that did not fit these frameworks were developed inductively to capture emergent patterns in participant accounts.

Third, codes were clustered into candidate themes and then reorganized around the emerging prototheory of tuning. This process involved moving iteratively between participant accounts, analytic memos, sensitizing theoretical constructs, and conceptual mapping. The first author conducted the initial coding and memoing. A second author independently reviewed a subset of the data and met with the first author to compare interpretations. A third author served as an analytic auditor and reviewed the developing code structure, candidate themes, and conceptual model, and all authors discussed these ongoing processes and interpretations during virtual meetings. During these meetings, the team discussed alternative interpretations, points of tension, and participant accounts that did not fit the emerging model. Analytic decisions were documented through code summaries, memos, and conceptual mapping. These procedures were used to distinguish participant accounts from sensitizing theoretical assumptions and to challenge coherence, fit, and potential overreach in the emerging prototheory.

Fourth, the team developed a thematic and conceptual map linking the qualitative themes to the recursive loops of the tuning prototheory. Illustrative quotes were selected to show how participant accounts supported, complicated, or bounded each component of the model. Counts are reported descriptively where useful to characterize the exit survey corpus, but they are not used to estimate prevalence in the broader BBB participant group.

Descriptive statistics were calculated only to characterize the available participant information, implementation participation, survey-section completion, and overall guided-experimentation rating. Available demographic variables were summarized using frequencies and percentages, and the guided-experimentation rating was summarized using the mean and number of respondents. These statistics were not used to evaluate intervention effectiveness or integrated with the qualitative coding.

### Research Team, Positionality, and Reflexivity

The analytic team included researchers with expertise in behavioral science, human-computer interaction, qualitative methods, health technology design, and XB/inbodied interaction. Several authors had prior involvement in the conceptual development, design, and/or implementation of XB. This provided deep contextual knowledge of the intervention and its theoretical aims but also created the possibility of confirmatory interpretation.

In the XB implementation, investigators also played an active facilitative role. They communicated with participants about group activities, responded to questions in the Teams space, posted prompts to stimulate discussion, and helped participants understand and prepare for the weekly experiments. Because investigators helped facilitate XB while also contributing to analysis, the research team occupied a dual role as both intervention facilitators and interpreters of participant accounts. This resembles broader qualitative discussions of insider-outsider positioning, membership roles, and reflexivity in interpretive inquiry [26-30].

### Trustworthiness

We addressed credibility through repeated engagement with the qualitative corpus, comparison of candidate themes across participant accounts, and attention to limiting or disconfirming cases. Dependability was supported through documentation of analytic decisions, code development, and conceptual mapping. Confirmability was supported through reflexive memoing, team-based review, and explicit distinction among participant accounts, sensitizing theoretical constructs, and author interpretation. Transferability was supported by providing a detailed description of the intervention context, recruitment approach, available participant characteristics, data sources, and analytic limitations.

### Reporting Guideline

We used the Standards for Reporting Qualitative Research (SRQR) [31] to guide reporting of the study rationale, qualitative approach, research team characteristics, sampling, data collection, analysis, findings, and limitations. Because the present study was a secondary, post hoc qualitative inquiry of intervention-generated participant accounts rather than a prospective interview or focus group study, SRQR was selected as the primary reporting guideline. Relevant domains from the Consolidated Criteria for Reporting Qualitative Research (COREQ) [32], particularly those related to research team characteristics, reflexivity, study context, sampling, and analysis, were also considered. A completed SRQR checklist is provided as Checklist 1.

### Ethical Considerations

This study was reviewed and approved by the University of Southampton Faculty of Environmental and Physical Sciences Ethics Committee (reference number: ERGO/FEPS/76885; submission version: 6, dated October 17, 2022). All participants were provided with a detailed participant information sheet outlining the purpose of the study, the voluntary nature of participation, the procedures involved, potential risks and benefits, and data handling practices. Informed consent was obtained from all participants prior to their involvement in the study. Data collected included limited nonidentifiable demographic and experiential information related to participants’ engagement with MOVE and EAT experiments. No sensitive health data were collected. Participant confidentiality was maintained by anonymizing data and storing personal information securely, in accordance with the university’s data protection policy and applicable data protection laws. Participants had the option to engage in a Teams group for discussion and support, with full transparency about data visibility in that setting. No participant compensation was provided. Participants retained the right to withdraw at any time without consequence.

## Results

### Participant Characteristics and App Rating

Available demographic information was limited to variables collected during the original BBB implementation. The original dataset included a binary female/male variable but did not clearly specify whether this referred to sex assigned at birth or gender identity. We therefore report this variable descriptively as collected and do not interpret it as either sex assigned at birth or gender identity. In the original BBB dataset, 24 participants (54.5%) were recorded as “female.” Additional participant characteristics, such as age, education, job role, race/ethnicity, or socioeconomic position, were not available for the present analysis. Among all participants who completed an exit survey (n=12), the average overall rating of the guided experimentation approach was 5.42 on a 1‐6 scale.

Because this analysis was conducted as a secondary, post hoc qualitative inquiry, participant counts are presented descriptively to characterize the available analytic corpus rather than to estimate prevalence in the broader BBB participant group. The qualitative findings below are grounded in participants’ open-text exit-survey responses.

### Overview of the Qualitative Themes

**Figure 1.**
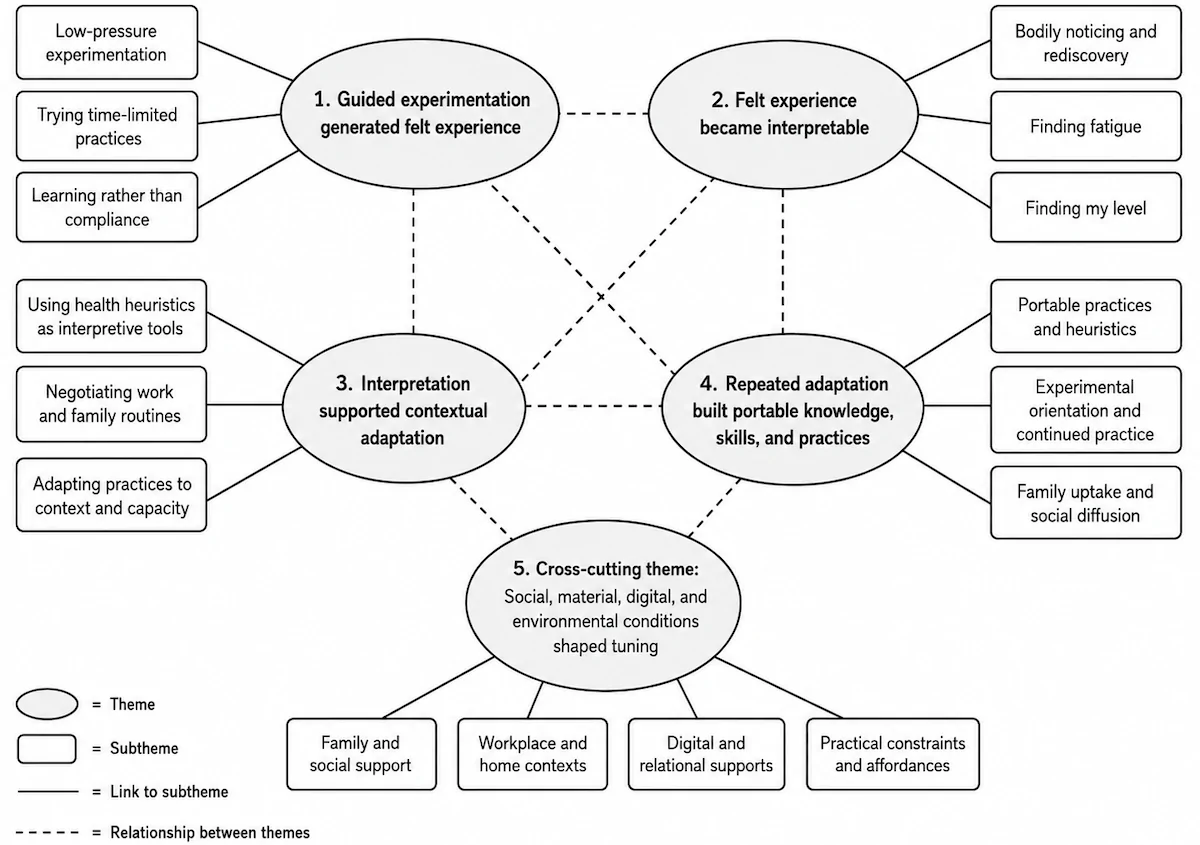
Thematic map of participant accounts of guided experimentation*.*

Reflexive thematic analysis identified four interrelated themes in participants’ accounts: (1) guided experimentation generated felt experience, (2) felt experience became interpretable, (3) interpretation supported contextual adaptation, and (4) repeated adaptation built portable knowledge, skills, and practices. Social, material, digital, and environmental conditions represented a crosscutting theme that could facilitate or constrain these processes. Figure 1 presents the relationships among the themes and subthemes. Through subsequent abductive analysis and conceptual mapping, the 4 themes were interpreted as recursive loops within the preliminary tuning prototheory. The sections below therefore present the empirical findings using the loop terminology of the resulting model.

### Overview of the Tuning Prototheory

**Figure 2.**
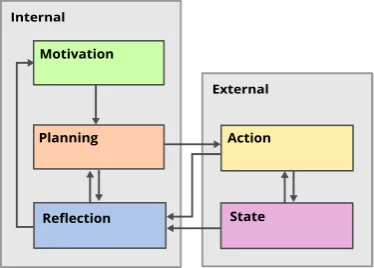
Visual overview of our tuning prototheory, comprising conceptual building blocks organized into 5 groups: motivation, planning, action, state, and reflection.

Through abductive analysis and conceptual mapping, the 4 empirical themes were organized into a preliminary prototheory of tuning. In this model, tuning is not a single behavior-change technique or psychological construct, but a dynamic process through which guided experimentation generates felt experience, reflection makes that experience interpretable, interpretation supports contextual adaptation, and repeated adaptation contributes to portable knowledge, skills, and practices. SDT and AT informed the interpretation of autonomy, competence, relatedness, tools, actions, and conditions, while the tuning prototheory foregrounds the recursive process through which felt experience becomes actionable self-knowledge. Figure 2 presents the conceptual building blocks of the prototheory.

### Loop 1: Guided Experimentation Generated Felt Experience

In the prototheory, each empirical theme corresponds to a recursive functional loop linking action, felt experience, interpretation, adaptation, and subsequent action. These loops are analytically distinguishable but operate as interdependent parts of the broader tuning process shown in Figure 2.

As represented in the action-to-state pathway in Figure 2, the first loop involved the relationship between guided experimentation and felt experience. Participants described XB as creating a low-pressure structure for trying eating and movement practices without requiring permanent commitment. Rather than being asked to adopt a diet or exercise program indefinitely, participants were invited to test short experiments, notice what happened, and decide whether and how to continue.

Several participants described this temporary and flexible structure as empowering. The experimental framing appeared to reduce the pressure often associated with health behavior change by making the task one of learning rather than compliance.

*It (XB) felt less daunting as the expectation was all about learning, trying it & then stopping in a few days if it didn’t suit you - I was surprised by how much I wanted to continue them beyond the 5 days.*

This account illustrates a key feature of tuning: the participant was not simply following instructions but generating experiential information. The short-term nature of the experiment created a safe opportunity to act, observe, and reassess. In SDT terms, this supported autonomy because participants could modify, continue, or discontinue practices based on their own experience. However, in the tuning prototheory, autonomy is not only a motivational state; it is part of a loop in which self-directed action creates felt experience that can then inform future action.

Participants also described the value of being able to find what worked for them rather than having a fixed behavior imposed on them. One participant described learning to adjust eating practices in a way that supported satisfaction and ongoing experimentation:

*I learnt a lot about how to manipulate my diet to get the satisfaction I needed for longer, alongside lots of tools to help me manage how I think about the choices I’m making. I’ve also started on my experiments with fermenting - some of which is going really well so far. I’m not sure if I & my family feel better for it (fermenting) yet, but the experiment is ongoing!*

This quote is especially important because it shows the loop continuing beyond the initial intervention. The participant describes not only a behavior change but also an experimental orientation: the experiment continues because the participant is still observing, interpreting, and adjusting.

### Loop 2: Felt Experience Became Interpretable

As represented in the state-to-reflection pathway in Figure 2, the second loop involved participants learning to notice and interpret bodily and experiential signals. Several participants reported that the MOVE experiments helped them reconnect with areas of the body they had neglected, underused, or not previously noticed. This was particularly visible in responses to flexibility, balance, hanging, coordination, and animal-movement experiments. Some participants described these experiences as a form of bodily rediscovery:

*I found these movement quite satisfying. Like stretching something you didn't know needed stretching.*

*The flexibility work on ankles & toes has been brilliant, and wasn't something that occurred to me to do before.*

*Its important to exercise all joints and muscles. My cycling and running had worked on muscle groups but i had neglected the others*.

These accounts suggest that XB did not only prompt participants to perform health behaviors. It invited them to notice how those behaviors were experienced. This noticing is central to tuning because felt experience becomes meaningful only when participants attend to it, compare it with prior assumptions, and begin to interpret it as information.

This process was also evident in participants’ descriptions of “finding my level.” Some participants reported that XB helped them identify forms or intensities of movement that matched their own capacities, limitations, and needs.

*It (XB) enabled me to find my level and be comfortable with each exercise.*

*It (XB) was useful because sometimes just saying “do this for 30 seconds” might be good for one person but not for another. Finding fatigue is unique to individuals.*

These responses show that participants were not only asking, “Did I complete the exercise?” but also, “What level is right for my body?” In the tuning prototheory, this marks the transition from action to interpretation. The participant acts, notices fatigue, comfort, satisfaction, or discomfort, and begins to use those signals to refine future engagement.

This loop extends beyond conventional adherence or performance metrics. Instead of treating success as completion of a prescribed task, participants described success as discovering a more personally appropriate relationship between action and felt bodily response.

### Loop 3: Interpretation Supported Contextual Adaptation

As represented in the reflection-to-planning pathway in Figure 2, the third loop involved the movement from interpretation to adaptation. Participants did not simply notice bodily signals; they used those signals, along with practical heuristics, to plan and adjust eating and movement practices to their lives. One way XB supported this interpretive process was by providing simple health heuristics. Participants described gaining knowledge and practical tools that helped them make sense of eating and movement choices. One participant described this as feeling “in the know”:

*All the tips on eating/exercising are easy and good to follow. It helped me feel that I am in the know and ahead of many peers that still follow “yesterday’s” outdated guidance.*

This quote reflects more than information acquisition. The participant describes a change in interpretive position: they feel better equipped to understand and act on eating and movement decisions. In the tuning prototheory, these heuristics function as psychological tools. They help participants connect felt experience with practical action.

However, adaptation was not only cognitive or motivational. It required participants to negotiate real-world constraints. This was especially evident in the contrast between MOVE and EAT experiments. Participants often described MOVE experiments as easier to integrate into daily routines, while EAT experiments required more planning, preparation, and coordination with work and family contexts.

*I did not find it (EAT experiments) difficult at all while I was at home. But I did not follow completely when I was in the office.*

*I did find the EAT part of this the hardest due to my circumstances, and not being organised enough to cook multiple meals for me and the family.*

*I found it easy to do this for the meals I eat alone/I prepare but not so easy for dinner especially if I wasn’t the one cooking.*

These accounts show that tuning is not a purely internal process. Participants could notice and interpret what might help them feel better, but whether they could act on that interpretation depended on available food, household routines, work schedules, and whether they controlled meal preparation. In AT terms, action was shaped by tools, conditions, and context. In the tuning prototheory, these contextual features are not background variables; they are part of the feedback loop that determines whether interpretation can become adaptive action.

Participants also described adapting movement practices to social and physical settings. Some found the animal movements playful and meaningful at home, especially with children:

*I think the animal moves will help me bond with my children and give them a good foundation in enjoying exercise.*

*I love the different approaches and trying new things I wouldn't otherwise think to do.*

However, the same movements could become difficult or uncomfortable in other settings:

*I enjoyed the playful side of the movements too, but have still not been brave enough to break into a “bear” in the office!*

*Animal moves...I felt uncomfortable doing these outside and struggled to find space at home to do these*.

These contrasting accounts are important because they show that the same experiment could be enabling or constraining depending on context. Tuning therefore requires not only bodily awareness but also contextual judgment: participants had to determine where, when, how, and with whom a practice could realistically fit.

### Loop 4: Repeated Adaptation Built Portable Knowledge, Skills, and Practices

As represented in the return from planning to future action in Figure 2, the fourth loop involved the accumulation of repeated experimentation into portable knowledge, skills, and practices. Many participants described intending to continue using heuristics or practices developed during BBB. These included movement snacks, flexibility practices, breathing-related movement practices, red/green food heuristics, finding fullness, and overnight fasting.

*The experiment has been really interesting & i intend to continue using the information I have learnt.*

*Yes definitely saw an improvement and will continue to monitor*.

Movement snacking appeared especially salient. Participants described small bursts of activity as easier to integrate into existing routines, including work routines. For some, this challenged the assumption that meaningful physical activity required longer or more intense exercise sessions. In the tuning prototheory, this represents a shift from externally defined exercise ideals toward personally usable practices that can be adapted across contexts.

Social diffusion also appeared in participant accounts. Several participants reported sharing experiments with family members or needing family members to understand the practices for them to be sustainable.

*But even my husband has noticed how much better he sleeps when we ensure we eat earlier, so it’s one we try really hard to stick to.*

*Need to educate and share the idea with my young adult children and husband, so they understand why i am doing what i am doing*.

These examples suggest that tuning can extend beyond the individual participant. Although XB was framed as an individual-guided experimentation process, participants often enacted it within family systems. When family members noticed benefits or supported the practice, the loop became easier to sustain. When family routines conflicted with the practice, adaptation became more difficult.

Over time, participants’ accounts suggest that tuning may become portable when the person carries forward not only a specific behavior but also a way of experimenting. The participant who wrote that “the experiment is ongoing” illustrates this shift. The practice is no longer only an assigned XB task; it has become a self-directed mode of inquiry.

### Crosscutting Theme: Social, Material, and Environmental Conditions Shaping Tuning

Across the 4 loops, participants’ exit-survey responses indicated that experimentation was shaped by the social, material, digital, and environmental conditions in which it occurred. Responses to the XB app and implementation questions contributed particularly to this crosscutting theme by describing how weekly meetings, Teams-based materials, investigator guidance, videos, family settings, work environments, available space, and food-preparation responsibilities facilitated or constrained participants’ ability to try, interpret, and adapt practices. These implementation-focused responses did not form a separate usability theme; rather, they helped explain how tools and relational supports mediated the tuning process.

Several participants described the weekly catch-up sessions and Teams interactions as useful for learning, accountability, and preparation:

*I enjoyed getting to listen to other people’s experiences of the study. I also liked having the meetings to discuss the upcoming weeks activities*.

*Communication on Teams was easy and effective, videos with movement suggestions were really useful, some challenges like sit/stand are fun and I can see the progress as I practice.*

These accounts suggest that social support did not operate only as encouragement. It also helped participants understand the experiments, prepare and plan for them, and interpret what they were experiencing. In SDT terms, these interactions may have supported relatedness and competence. In the tuning prototheory, they also helped sustain the recursive process by keeping participants engaged with the tools, practices, and interpretive resources needed for experimentation.

The social environment was especially important when participants tried to integrate experiments into family life. For some, family became a site of support and shared discovery. For others, family routines introduced constraints, especially around EAT experiments. Similarly, the workplace could support some practices, such as movement snacks, while making others socially uncomfortable or logistically difficult.

Taken together, these findings suggest that tuning unfolds within an ecology of support and constraint. The participant is not simply choosing whether to engage. They are continually negotiating the fit between bodily experience, practical tools, social expectations, available resources, and environmental affordances.

### Core Recursive Loops of the Tuning Prototheory

**Figure 3.**
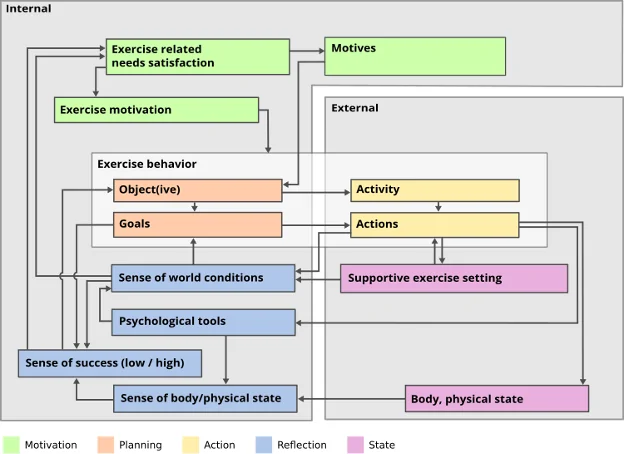
Detailed overview of our tuning prototheory comprising conceptual building blocks derived from our qualitative abductive analysis.

Taken together, these findings support a prototheory of tuning composed of interlocking feedback loops. These loops are not linear stages. Rather, motivation leads to experimentation; experimentation generates embodied outcomes; reflection turns those outcomes into insight; insight refines future action; and repeated action builds confidence, skill, and contextual knowledge. Figure 3 provides a detailed overview of these recursive relationships.

Although SDT and AT helped interpret major parts of this process, the tuning prototheory adds an explicit focus on how felt experience becomes actionable self-knowledge. SDT helped explain how autonomy, competence, and relatedness supported engagement. AT helped explain how tools, actions, objects, and conditions shaped what participants could do. However, neither theory fully accounted for the recursive process through which participants used guided experimentation to sense, interpret, adapt, and carry forward practices based on felt experience. Tuning therefore names the dynamic process by which participants learned to live and act in closer alignment with their bodies, goals, and lived realities.

### Boundary Conditions and Limiting Cases

The data also revealed important boundary conditions. First, not all experiments were equally easy to integrate. EAT experiments often required more preparation than MOVE experiments and were more vulnerable to work schedules, food availability, household routines, and whether participants controlled meal preparation. Second, some MOVE practices, particularly animal movements, were experienced as playful and engaging in some contexts but uncomfortable or impractical in others. Third, social context could either support or constrain tuning: family involvement sometimes strengthened engagement, but family routines could also make experimentation more difficult.

Finally, the primary qualitative corpus came from exit survey respondents. These accounts may overrepresent participants who were more engaged, had more positive experiences, or were more motivated to provide feedback. Because the present analysis was secondary and post hoc, systematic information about reasons for nonresponse or noncompletion was limited. These boundary conditions informed the prototheory by emphasizing that tuning is not simply an individual psychological capacity. It is a situated process shaped by social, material, environmental, and interpretive conditions.

## Discussion

### Overall Findings

This secondary, post hoc interpretive qualitative analysis developed a preliminary prototheory of tuning from participant accounts of the BBB 2023 implementation of XB. The central finding is that participants did not describe XB only as a set of eating and movement tasks, but as a guided process for trying practices, noticing felt experience, interpreting bodily and contextual feedback, adapting actions, and carrying forward personally meaningful knowledge, skills, and practices. We conceptualize this process as tuning: a recursive, person-centered process through which felt experience becomes actionable self-knowledge.

### Theoretical Contribution

The prototheory contributes to behavior change and digital health research by foregrounding the recursive relationship among action, felt experience, interpretation, and adaptation. Many intervention models emphasize motivation, adherence, self-monitoring, or external feedback. In contrast, tuning highlights how participants may learn from internally sensed and contextually situated experience. In this account, the body is not only the target of behavior change but also a source of feedback that participants learn to notice and use.

### Relationship to SDT and AT

SDT helped explain why XB may have supported engagement: the intervention structure appeared to support autonomy by allowing participants to choose, modify, and discontinue experiments; competence by providing practical heuristics and skills; and relatedness through group discussion, family involvement, and investigator support. AT helped explain how actions were mediated by tools, conditions, and social/material contexts. However, the prototheory of tuning extends these frameworks by focusing on the recursive process through which participants use felt experience to refine future action. Thus, SDT and AT help explain important parts of the system, while tuning names the dynamic process by which those parts become experientially integrated.

### Implications

#### Implications for Digital Health Design

The tuning prototheory suggests that digital health tools designed to support eating, movement, and other health practices should not only prompt behavior, track outcomes, or deliver reminders. They should also help users generate interpretable experiences. This aligns with prior work on XB, tuning, and inbodied interaction, which positions felt experience and bodily awareness as central to healthful adaptation [9-12]. In XB, this occurred through short, low-risk experiments, practical heuristics, reflection prompts, and opportunities to adapt practices to bodily, social, and environmental conditions.

A tuning-oriented design approach is also consistent with work on interoception and body sense, which emphasizes that bodily signals must be sensed, interpreted, and integrated before they can guide action [1-3]. Rather than treating internal experience as secondary to external metrics, tuning-oriented tools could help users connect practices with felt changes in energy, fullness, fatigue, comfort, or discomfort. These tools may therefore be especially useful when the design goal is not simply adherence to a prescribed behavior but the development of portable knowledge, skills, and practices that users can adapt beyond the intervention.

This framing contrasts with some behavior-change technologies that rely primarily on external prompts, persuasion, reminders, rewards, or nudges to shape behavior [33-35]. Such approaches can be useful in some contexts, including clinical or public health contexts, but they also raise ethical and practical questions about autonomy, paternalism, dependence on external cues, and the durability of behavior change [36-39]. Tuning offers a complementary design orientation: rather than positioning the system as the expert that directs behavior, the system scaffolds users’ capacity to experiment, interpret experience, and adapt practices in ways that feel personally and contextually meaningful.

#### Implications for Relational and Social Support

The findings also highlight the importance of relational support in guided experimentation. In their exit-survey responses, participants described value in weekly meetings, Teams-based materials, investigator guidance, and family involvement. These social interactions appeared to support not only accountability but also interpretation, preparation, and contextual adaptation. In SDT terms, these interactions may support relatedness and competence by helping participants feel connected, capable, and supported in their experimentation [18-21]. In the tuning prototheory, relational support also helps sustain the recursive process by keeping participants engaged with the tools, practices, and interpretive resources needed for experimentation.

This has implications for how tuning-oriented interventions are designed and scaled. In XB, investigators helped explain experiments, answer questions, and facilitate reflection. These activities may have supported participants’ ability to interpret and adapt their experiences, but they also raise practical questions about which forms of guidance are essential and which could be distributed among peers, families, community facilitators, or digital tools. Future implementations should examine how relational support can be provided in ways that preserve autonomy, avoid overdirecting participant interpretation, and support community ownership of the experimentation process.

#### Implications for Equity and Trauma-Informed Design

Although the present study was conducted with university staff and should not be generalized to populations not represented in the sample, the tuning framework may have relevance for future work with people who experience difficulty accessing, trusting, or acting on bodily cues. Prior work suggests that trauma exposure, chronic stress, emotional distress, and adverse social conditions can shape how people experience, interpret, or disconnect from bodily signals [4,5,40]. In some contexts, disconnection from bodily sensation may function as a protective or adaptive response rather than a simple deficit [4,5]. This is important for digital health design because interventions that assume users can easily notice, trust, or act on internal cues may not be appropriate for all populations.

A tuning-oriented approach may offer a gentler alternative by treating bodily awareness as something that can be gradually cultivated through choice, experimentation, reflection, and contextual adaptation. Rather than asking users to comply with externally imposed goals, tuning-oriented systems could support users in rebuilding relationships with bodily experience at a pace and intensity that preserves autonomy. This emphasis also aligns with ethical concerns about autonomy, vulnerability, and persuasive technologies, particularly for people or communities whose agency has been constrained by structural inequities or prior harmful experiences with health systems [41,42]. Future studies should examine whether guided experimentation can be adapted for trauma-informed and equity-oriented digital health contexts while avoiding assumptions about participants’ needs, capacities, or relationships to their bodies.

### Limitations

This study has several limitations. First, the analysis was conducted as a secondary, post hoc inquiry using data generated during a real-world implementation rather than a prospectively designed qualitative study. As a result, available demographic information, reasons for nonparticipation, reasons for exit survey noncompletion, and systematic follow-up with nonrespondents were limited. Second, the primary qualitative corpus consisted of open-text exit survey responses from participants who completed the postintervention survey, which may overrepresent participants who were more engaged, more satisfied, or more motivated to provide feedback. Claims regarding participant experience are grounded in the open-text exit-survey responses. App interaction records were used only to characterize implementation participation, and the underlying Teams discussions and meeting recordings were not analyzed. Third, several authors were involved in the development and/or implementation of XB, which provided contextual insight but also introduced the possibility of confirmatory interpretation. We addressed this through reflexive memoing, team-based review, attention to limiting cases, and explicit distinction between participant accounts, sensitizing theory, and author interpretation. Fourth, because prior positive outcomes or side effects of XB were mentioned to participants, expectancy effects may have shaped some participant accounts. Finally, the prototheory should be treated as preliminary and generative rather than definitive.

### Future Work

Future research should prospectively design qualitative and mixed methods studies to test, refine, and potentially challenge the tuning prototheory. Such work should include more systematic sampling, richer participant characterization, interviews or focus groups designed specifically to examine tuning processes, longitudinal follow-up, and explicit examination of participants who disengage or do not experience benefit. Future studies should also examine whether tuning can be operationalized through measurable constructs, such as interoceptive awareness, perceived autonomy, contextual fit, self-efficacy, and sustained use of personally adapted health heuristics.

Future research should also examine the facilitator role in tuning-oriented interventions. In the present XB implementation, investigators helped participants understand the experiments, answered questions, and supported reflection. Future studies should examine which aspects of this relational guidance are essential to tuning, which can be distributed among peers or community members, and which can be supported by digital tools without undermining autonomy or overdirecting interpretation. This is especially important for scaling XB-like approaches while preserving the relational and interpretive support that participants appeared to value.

### Conclusions

This study developed a preliminary prototheory of tuning to explain how participants used guided experimentation to connect action, felt experience, interpretation, and adaptation. The theory suggests that XB may support behavior change not only by encouraging eating and movement practices, but by helping participants cultivate the capacity to notice, interpret, and act on felt experience. Tuning offers a generative framework for designing and studying digital health tools that support personally meaningful, context-sensitive, and sustainable health behavior change.

## Supplementary material

10.2196/85164 Multimedia Appendix 1 XB application screenshots.

10.2196/85164 Checklist 1 SRQR checklist.

## Abbreviations:

AT: activity theory
BBB: Build Burn Better
COREQ: Consolidated Criteria for Reporting Qualitative Research
SDT: self-determination theory
SRQR: Standards for Reporting Qualitative Research
TCM: theory construction methodology
XB: Experiment in a Box
