# Supplementary material for: Learning the Capacity to Feel Better Through Experiment in a Box (XB): Qualitative Interpretive Description and Prototheory Development of Tuning
Source: JMIR Form Res. 2026 Sep 21;10:e85164. doi: 10.2196/85164 (PMC13592457; doi:10.2196/85164)
Supplement: Supplementary file 1 [file formative-v10-e85164-s001.pdf]

18:56 4G

/labs/overview

‹ Back to Labs

Welcome to the Build Burn Recover Better Study

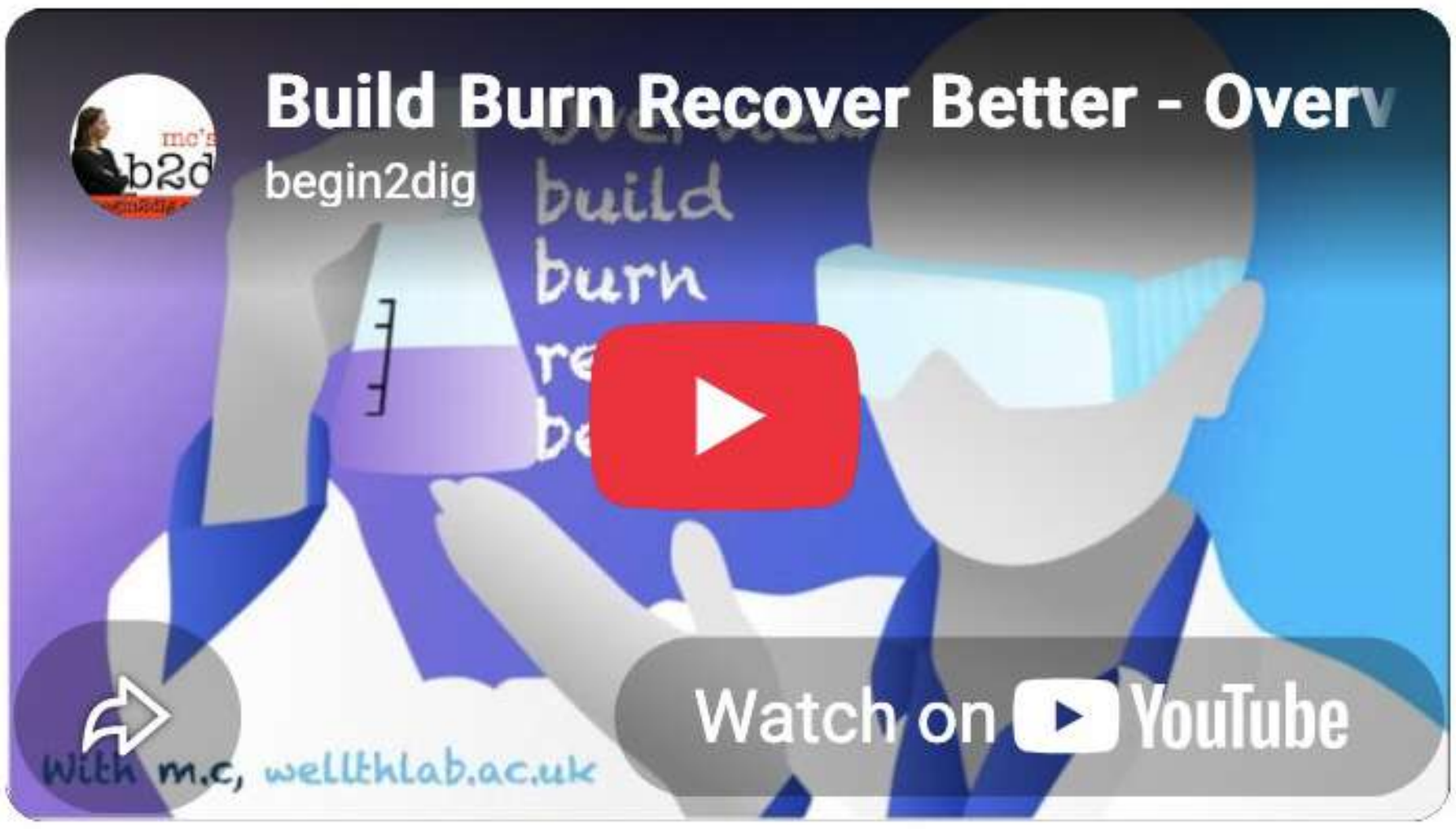

Goal: over the next four weeks, to Explore some fundamentals about Move, Eat and Sleep - so you can build real experience, knowledge skills and practice of how to feel better - anytime, anywhere, forever. Really.

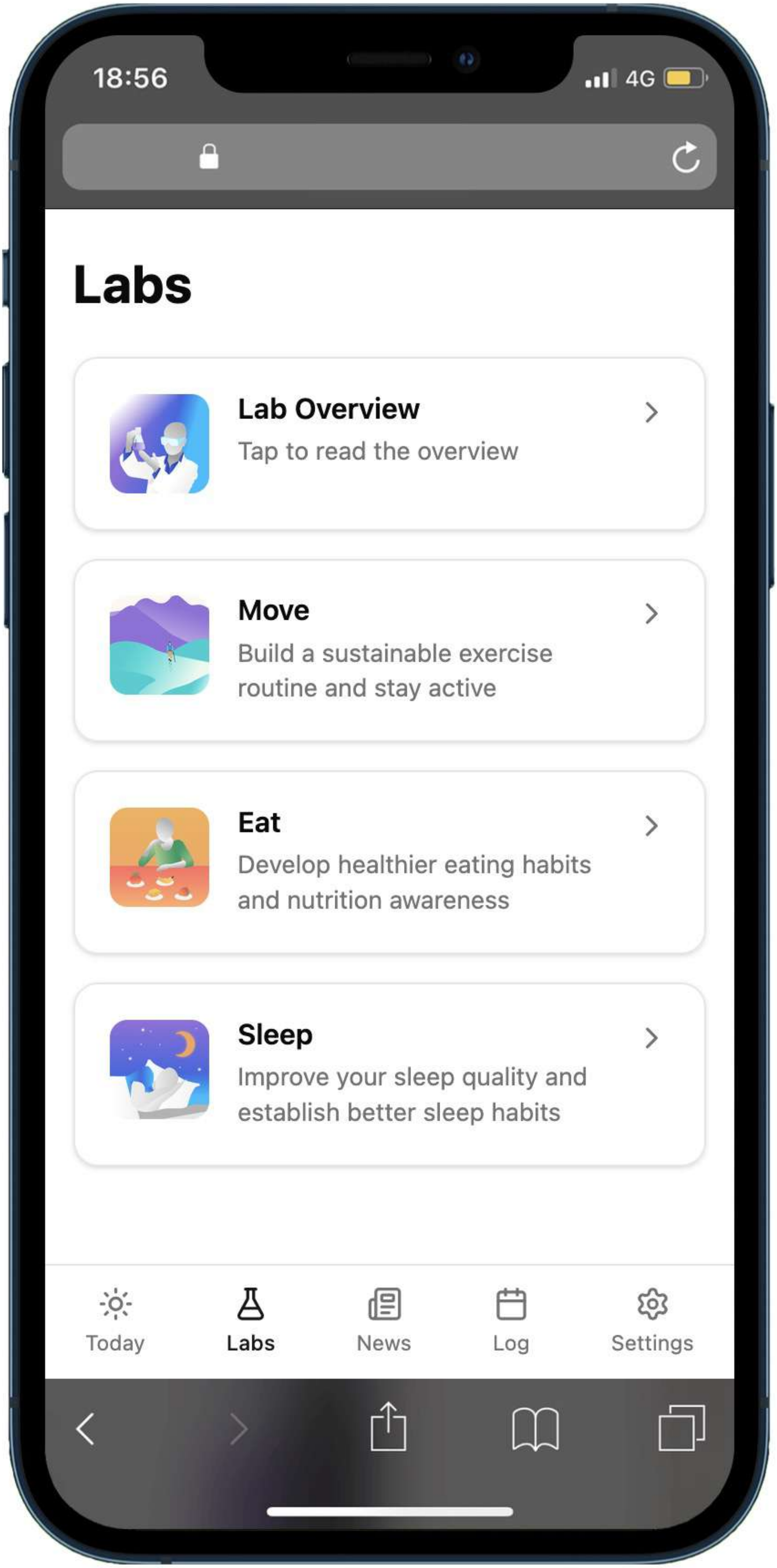
18:56
4G
Labs
Lab Overview
Tap to read the overview
Move
Build a sustainable exercise routine and stay active
Eat
Develop healthier eating habits and nutrition awareness
Sleep
Improve your sleep quality and establish better sleep habits
Today
Labs
News
Log
Settings

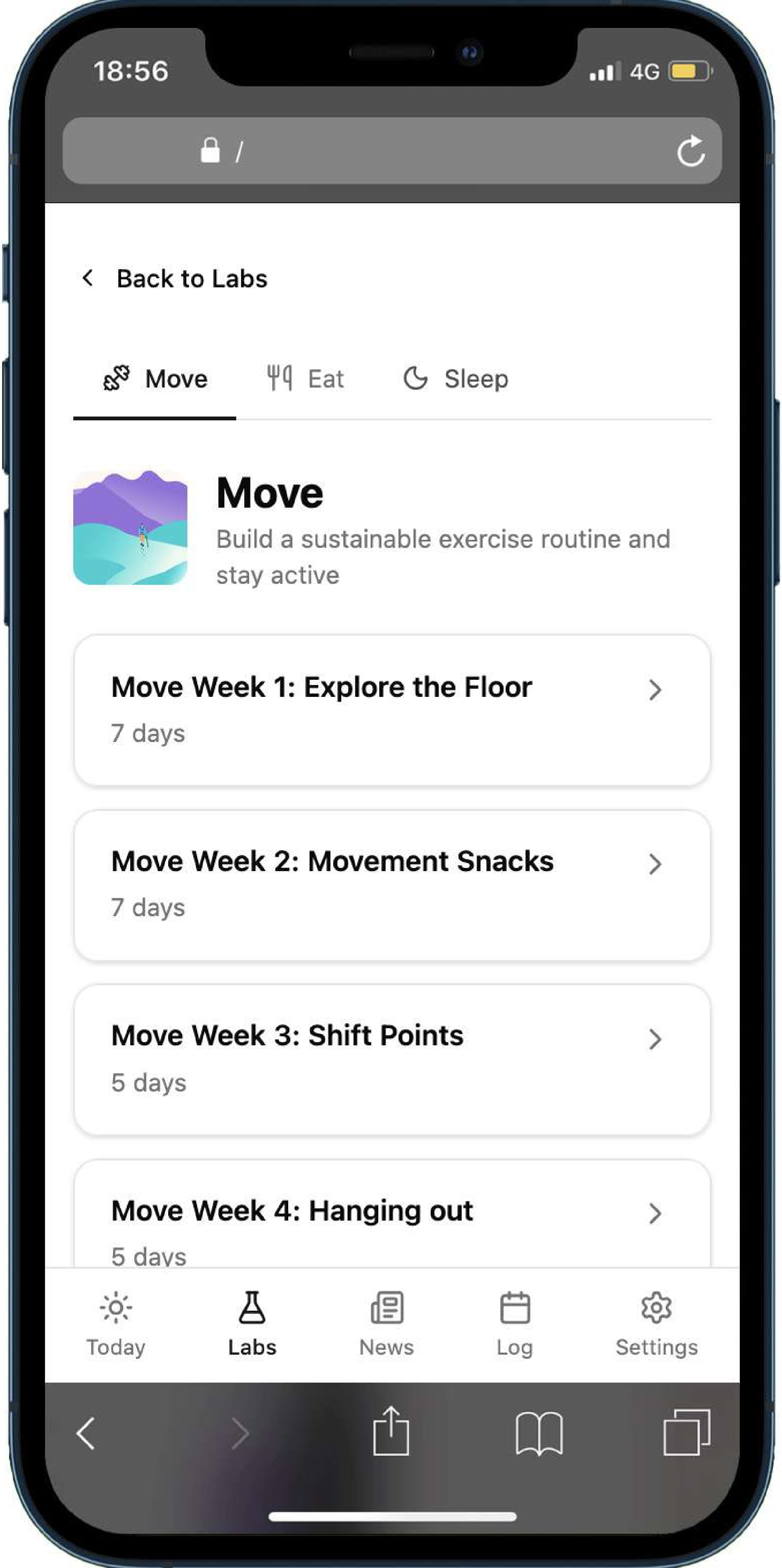
18:56
4G
/
Back to Labs
Move
Eat
Sleep
Move
Build a sustainable exercise routine and stay active
Move Week 1: Explore the Floor
7 days
Move Week 2: Movement Snacks
7 days
Move Week 3: Shift Points
5 days
Move Week 4: Hanging out
5 days
Today
Labs
News
Log
Settings

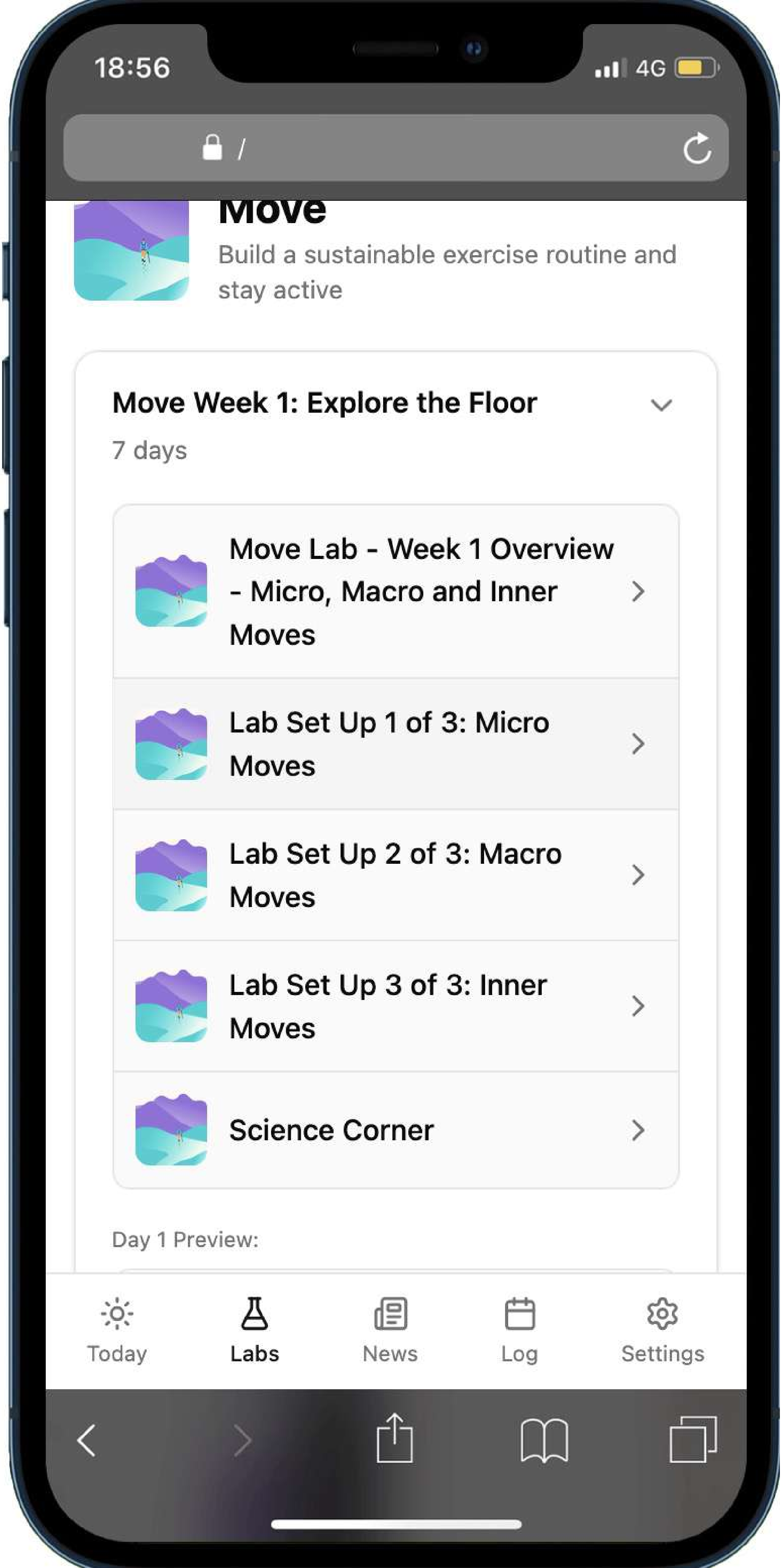
18:56
4G
/
Move
Build a sustainable exercise routine and stay active
Move Week 1: Explore the Floor
7 days
Move Lab - Week 1 Overview - Micro, Macro and Inner Moves
Lab Set Up 1 of 3: Micro Moves
Lab Set Up 2 of 3: Macro Moves
Lab Set Up 3 of 3: Inner Moves
Science Corner
Day 1 Preview:
Today
Labs
News
Log
Settings

18:56 4G

/

Move

Build a sustainable exercise routine and

Overview

This week's focus is to see how simple things like focusing on **how we can connect with the ground** can help us build strength and balance anytime, anywhere.

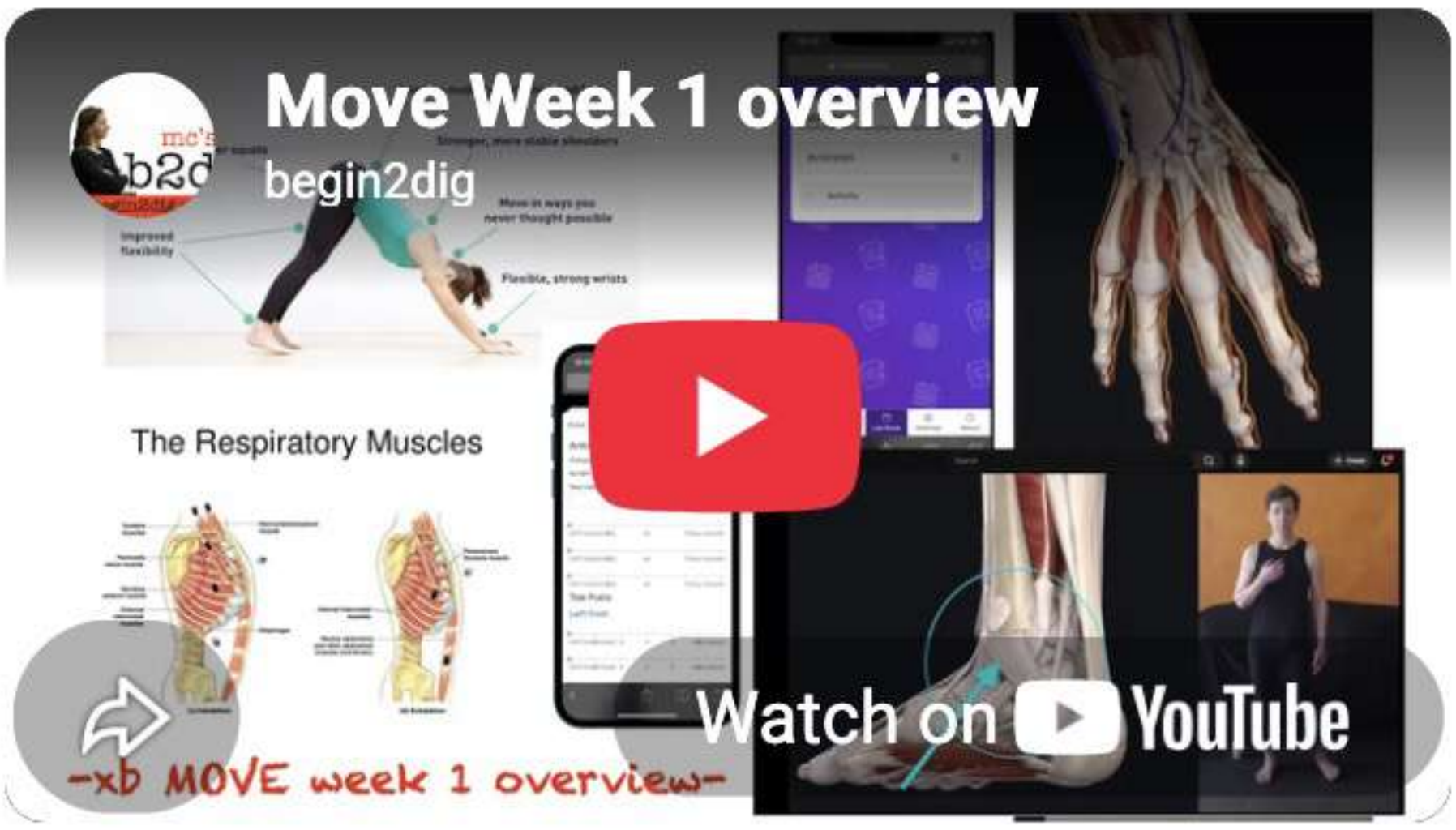

Ground Control: Your focus in this week's experiment is all about considering how you feel when moving - from small movements to bigger ones.

Close

18:56 4G

Move Week 1: Explore the Floor

7 days

Lab set up

TARGETS for this experiment are: to move to feel a small pull/movement right under the ankle bone - the malleoli - both inside the foot and the outside the foot movement. See where the arrows are pointing in this image:

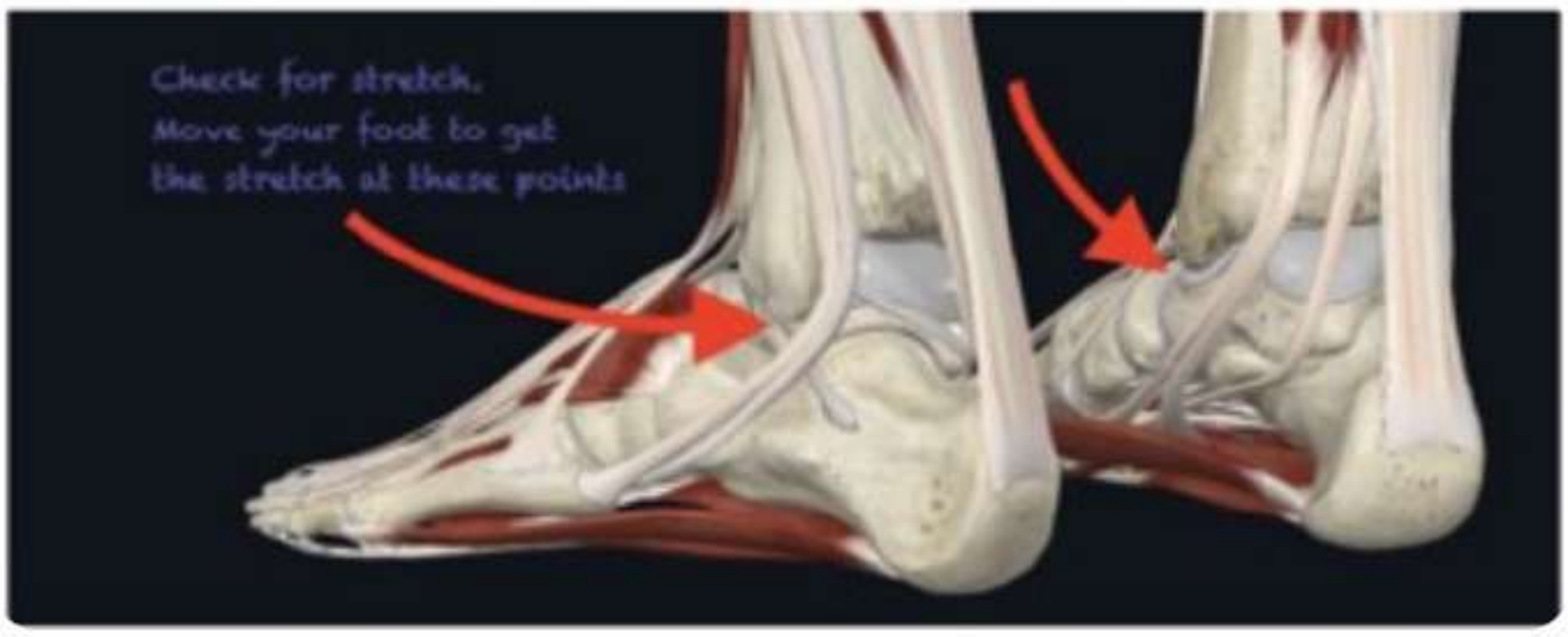

Please watch the following video for a demonstration of the moves:

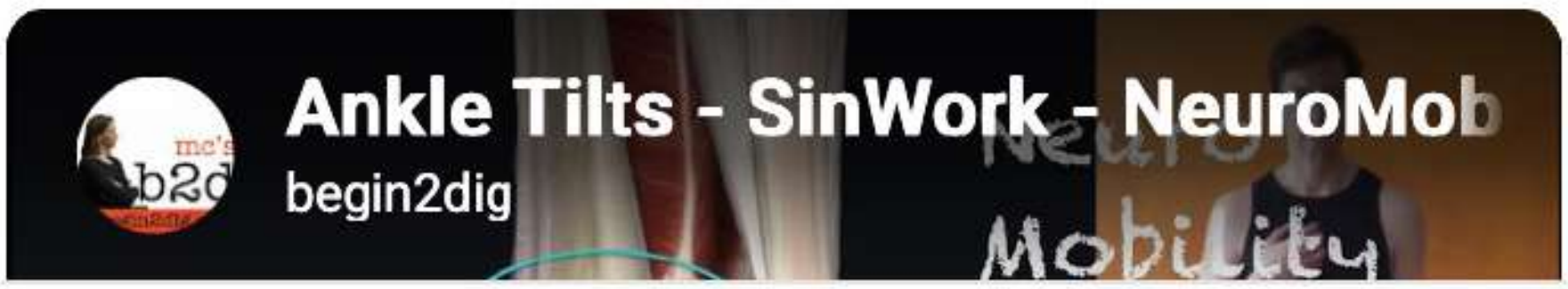

Close

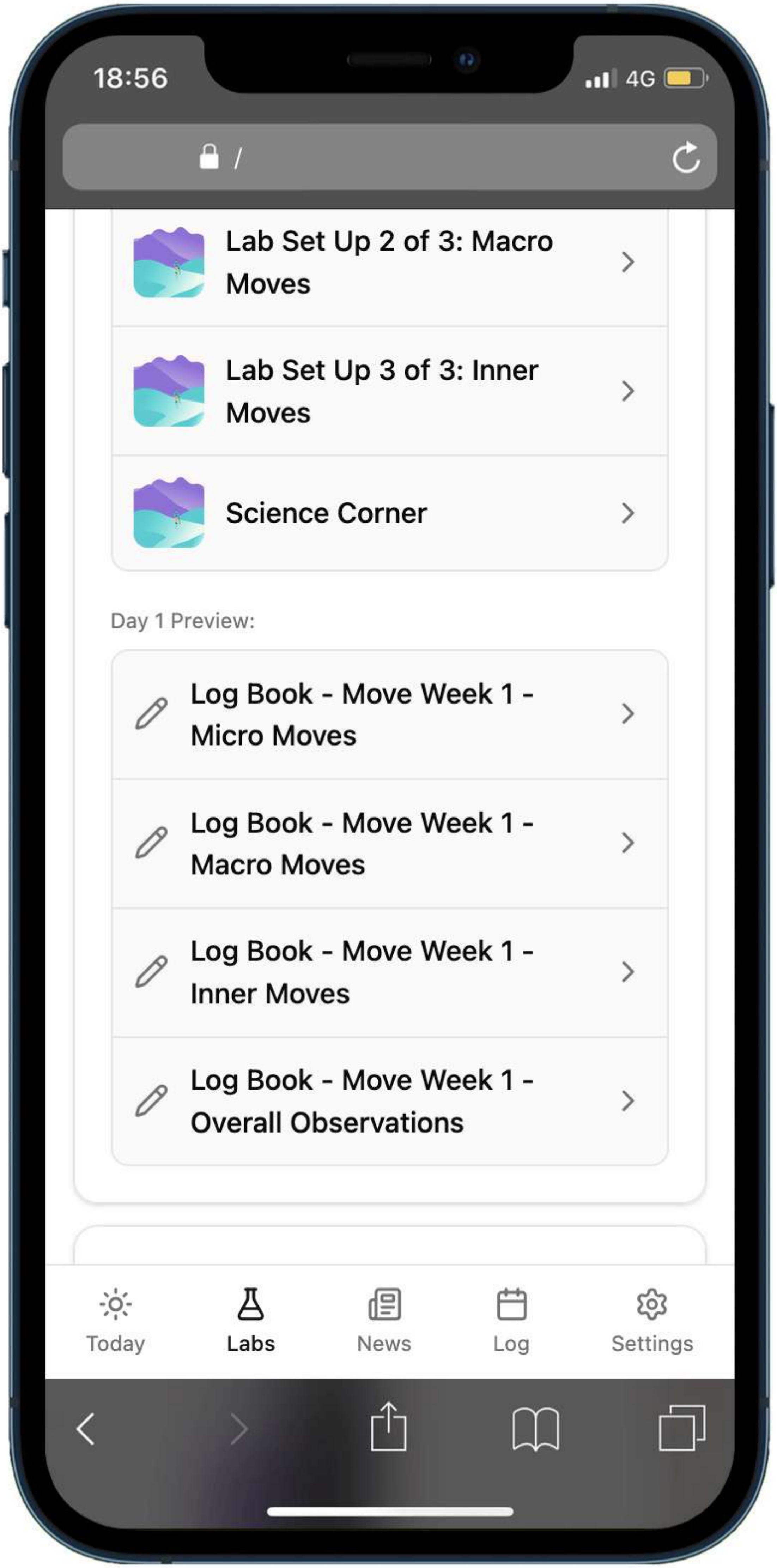
18:56
4G
/
Lab Set Up 2 of 3: Macro Moves
Lab Set Up 3 of 3: Inner Moves
Science Corner
Day 1 Preview:
Log Book - Move Week 1 - Micro Moves
Log Book - Move Week 1 - Macro Moves
Log Book - Move Week 1 - Inner Moves
Log Book - Move Week 1 - Overall Observations
Today
Labs
News
Log
Settings

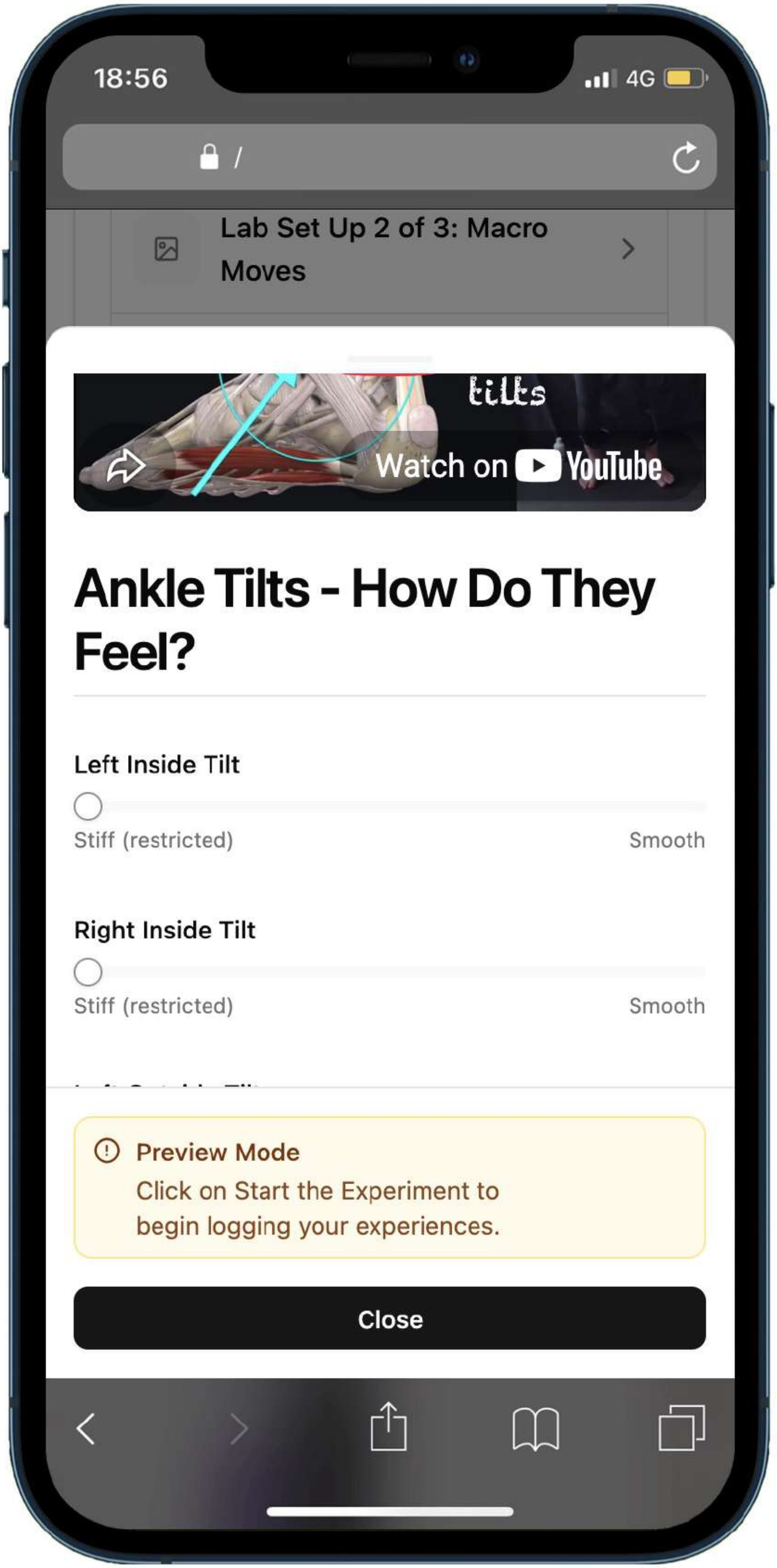
18:56
4G
/
Lab Set Up 2 of 3: Macro Moves
tilts
Watch on YouTube
Ankle Tilts - How Do They Feel?
Left Inside Tilt
Stiff (restricted)
Smooth
Right Inside Tilt
Stiff (restricted)
Smooth
Preview Mode
Click on Start the Experiment to begin logging your experiences.
Close
