# Supplementary material for: Learning the Capacity to Feel Better Through Experiment in a Box (XB): Qualitative Interpretive Description and Prototheory Development of Tuning
Source: JMIR Form Res. 2026 Sep 21;10:e85164. doi: 10.2196/85164 (PMC13592457; doi:10.2196/85164)
Supplement: Supplementary file 2 [file formative-v10-e85164-s002.docx]

**Standards for Reporting Qualitative Research (SRQR) Checklist**

This checklist was completed to support transparent reporting of the qualitative components of the manuscript. Because the study was a secondary, post hoc qualitative inquiry using intervention-generated open-text participant accounts rather than a prospective interview or focus group study, the Standards for Reporting Qualitative Research (SRQR) was used as the primary reporting guideline. Relevant COREQ domains related to researcher characteristics, reflexivity, context, sampling, and analysis were also considered in the manuscript revision.

**Study design:** Secondary, post hoc qualitative inquiry using interpretive description, reflexive thematic analysis, and abductive analysis

**Primary qualitative corpus:** Open-text postintervention survey responses from Build Burn Better 2023 participants, comprising all nonblank responses to 39 open-text questions across 3 survey sections, provided by up to 12 participants.

| **SRQR Item** | **Description** | **Reported in Manuscript** | **Location** |
| --- | --- | --- | --- |
| 1. Title | Concise description of the nature and topic of the study, identifying the study as qualitative or indicating the approach | Yes | Page 1: Title |
| 2. Abstract | Summary of key elements: background, purpose, qualitative approach, methods, results, and conclusions | Yes | Pages 1-2: Abstract |
| 3. Problem formulation | Description and significance of the problem or phenomenon studied | Yes | Page 2: Introduction - Background; Experiment in a Box (XB) and Tuning |
| 4. Purpose or research question | Purpose of the study or research question | Yes | Page 2: Introduction - Objective |
| 5. Qualitative approach and research paradigm | Qualitative approach, theory, or methodology identified and justified | Yes | Pages 2-3: Methods - Study Design and Methodological Orientation |
| 6. Researcher characteristics and reflexivity | Researcher characteristics that may influence the study, including prior relationships, assumptions, or involvement | Yes | Page 6: Methods - Research Team, Positionality, and Reflexivity |
| 7. Context | Setting/site and relevant contextual factors | Yes | Page 3: Methods - The XB Approach; Setting and Implementation Context; Participants, Recruitment, and Sampling |
| 8. Sampling strategy | How and why participants or data sources were selected | Yes | Page 3: Methods - Participants, Recruitment, and Sampling |
| 9. Ethical issues pertaining to human subjects | Ethics approval, consent, confidentiality, and participant protections | Yes | Page 6: Methods - Ethical Considerations |
| 10. Data collection methods | Types of data collected and procedures used | Yes | Pages 3-5: Methods - Data Collection; Table 1; Table 2 |
| 11. Data collection instruments and technologies | Instruments, technologies, platforms, or guides used for data collection | Yes | Pages 3-5: Methods - The XB Approach; Data Collection; Table 1; Table 2 |
| 12. Units of study | Number and relevant characteristics of participants, documents, or data units | Yes | Pages 3 and 6: Methods - Participants, Recruitment, and Sampling; Results - Participant Characteristics and App Rating |
| 13. Data processing | Methods for handling, preparing, and managing data prior to analysis | Yes | Pages 5-6: Methods - Data Analysis |
| 14. Data analysis | Analytic process, coding, theme development, theory development, and role of software | Yes | Pages 5-6: Methods - Data Analysis |
| 15. Techniques to enhance trustworthiness | Strategies for credibility, dependability, confirmability, and transferability | Yes | Page 6: Methods - Trustworthiness |
| 16. Synthesis and interpretation | Main findings, themes, concepts, or theory | Yes | Pages 6-11: Results - Overview of the Qualitative Themes; Overview of the Tuning Prototheory; Loop 1-4 sections; Core Recursive Loops of the Tuning Prototheory |
| 17. Links to empirical data | Use of quotations, examples, or other evidence to support findings | Yes | Pages 8-12: Results - Loop 1-4 sections; Crosscutting Theme: Social, Material, and Environmental Conditions Shaping Tuning; Boundary Conditions and Limiting Cases |
| 18. Integration with prior work, implications, transferability, and contribution | Relationship to existing literature/theory and implications | Yes | Pages 12-13: Discussion - Theoretical Contribution; Relationship to SDT and AT; Implications |
| 19. Limitations | Study limitations and constraints on interpretation | Yes | Page 13: Discussion - Limitations |
| 20. Conflicts of interest | Potential conflicts or declaration of none | Yes | Page 14: Conflicts of Interest |
| 21. Funding | Sources of funding and role of funder, if applicable | Yes | Page 14: Funding |
